# Identification of Spindle and Kinetochore-Associated Family Genes as Therapeutic Targets and Prognostic Biomarkers in Pancreas Ductal Adenocarcinoma Microenvironment

**DOI:** 10.3389/fonc.2020.553536

**Published:** 2020-11-02

**Authors:** Yi Liu, Zong-rui Jin, Xing Huang, Ye-cheng Che, Qin Liu

**Affiliations:** ^1^Department of Gastrointestinal Surgery, Guangxi Medical University Cancer Hospital, Guangxi Clinical Research Center for Colorectal Cancer, Nanning, China; ^2^Department of Hepatobiliary Surgery, The First Affiliated Hospital of Guangxi Medical University, Nanning, China; ^3^Department of Radiotherapy, The Second Affiliated Hospital of Guangxi Medical University, Nanning, China; ^4^Department of Emergency Medicine, First People’s Hospital of Fuzhou, Fuzhou, China; ^5^Department of Medical Ultrasonics, Second People’s Hospital of Guilin, Guilin, China

**Keywords:** biomarker, spindle and kinetochore associated, prognosis, pancreatic cancer, bioinformatics analysis, immune infiltration

## Abstract

**Aim:**

The role of spindle and kinetochore-associated (SKA) genes in tumorigenesis and cancer progression has been widely studied. However, so far, the oncogenic involvement of SKA family genes in pancreatic cancer and their prognostic potential remain unknown.

**Methods:**

Here, we carried out a meta-analysis of the differential expression of SKA genes in normal and tumor tissue. Univariate and multivariate survival analyses were done to evaluate the correlation between SKA family gene expression and pancreas ductal adenocarcinoma (PDAC) prognosis. Joint-effect and stratified survival analysis as well as nomogram analysis were used to estimate the prognostic value of genes. The underlying regulatory and biological mechanisms were identified by Gene set enrichment analysis. Interaction between SKA prognosis-related genes and immune cell infiltration was assessed using the Tumor Immune Estimation Resource tool.

**Results:**

We find that *SKA1–3* are highly expressed in PDAC tissues relative to non-cancer tissues. Survival analysis revealed that high expression of *SKA1* and *SKA3* independently indicate poor prognosis but they are not associated with relapse-free survival. The prognostic value of *SKA1* and *SKA3* was further confirmed by the nomogram, joint-effect, and stratified survival analysis. Analysis of underlying mechanisms reveals that these genes influence cancer-related signaling pathways, kinases, miRNA, and E2F family genes. Notably, prognosis-related genes are inversely correlated with several immune cells infiltrating levels.

**Conclusion:**

We find that *SKA1* and *SKA3* expression correlates with prognosis and immune cell infiltration in PDAC, highlighting their potential as pancreatic cancer prognostic biomarkers.

## Introduction

Pancreatic cancer is one of the most aggressive malignancies in the world and is associated with a high rate of metastasis and mortality. Its 5-year survival rate is estimated to be 5% globally, and 11.7% in China (1–3). It is estimated that in 2018, there were 458,918 new pancreatic cancer cases and 432,242 patient deaths worldwide (4). Pancreatic cancer management is mainly by surgical resection, which is thought to improve the 5-year survival to 20–30% (5). Non-etheless, surgical resections are recommended for <20% of pancreatic cancer cases since the disease is often diagnosed at the advanced stage (5–7). Therefore, strategies for early detection and treatment of pancreatic cancer are urgently needed.

It is well-known that mitosis is a common biological process in eukaryotic cells. During mitosis, the spindle ensures that sister chromatids are correctly distributed between daughter cells (8). *SKA1–3* family of genes are essential for the accurate timing of late mitosis. Spindle and kinetochore-associated (SKA) complex produced by the SKA genes maintain stability of metaphase plate or spindle checkpoint silencing (9, 10). Upregulation of *SKA1* triggers nucleation of interphase microtubules, while depletion of the *SKA1* complex results in abnormal mitosis (11, 12). The association between *SKA1* and cancer has been widely investigated. It has been reported that *SKA1* overexpression may lead to the development of pancreatic cancer in mouse models (13). *SKA1* upregulation has also been observed in multiple malignancies, including gastric, oral, bladder, non-small cell lung, hepatocellular, and prostate cancer. Elevated levels of *SKA1* have been shown to promote cancer cell proliferation and influence (14–20).

Given the limited treatment options for pancreas ductal adenocarcinoma (PDAC), novel biomarkers are needed to enhance treatment and prognosis. Previous reports show that SKA family genes may influence cancer treatment response and prognosis. We therefore speculated that SKA family genes might be prognostic in PDAC. To test this possibility, we used bioinformatics to investigate their expression in PDAC and how it correlates with prognosis.

## Materials and Methods

### Acquisition of Public RNA-Seq and Gene Microarray Data

**FIGURE 1:**
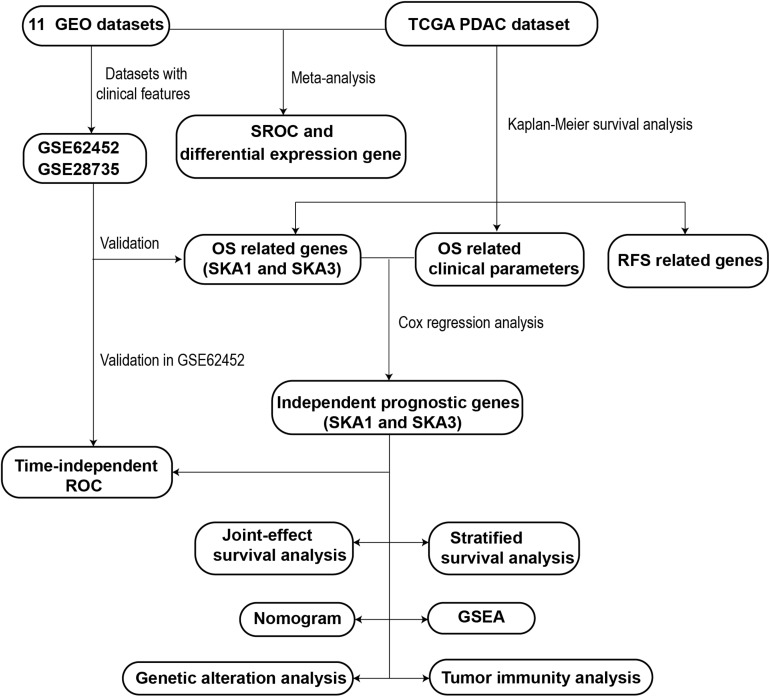
Flowchart presenting the general work flow of this study.

A schematic representation of our study outline is shown in Figure 1. Violin plots and RNA-seq data of SKA gene expression in normal tissues were obtained from Genotype-Tissue Expression (GTEx)^1^ (21, 22). RNA-seq raw data for 149 samples (145 PDAC tissue and 4 non-tumor pancreatic tissue) were obtained from The Cancer Genome Atlas (TCGA)^2^ (23). Matched clinical information of the PDAC patients was obtained from University of California Santa Cruz Xena Platform (UCSC Xena).^3^ RNA-seq raw data were normalized by DESeq package (24). Pancreas ductal adenocarcinoma SKA gene expression profile microarrays, normalized using limma package (25), were retrieved from Gene Expression Omnibus (GEO).^4^ All the expression profile data were log2-transformed before further analysis.

### Meta-Analysis

The following search terms were used to retrieve relevant datasets from GEO: “pancreatic cancer,” “mRNA,” and “pancreas.” The inclusion criteria for PDAC microarrays were as follows: (1) it must contain both normal and tumor samples, (2) expression profile data are available, (3) samples are all human, and (4) ≥10 samples available. A schematic of the search process is shown in Supplementary Figure 1. Details of the included microarrays are shown in Supplementary Table 1. Eleven previously published datasets were retrieved (GSE71729, GSE28735, GSE15471, GSE62165, GSE62452, GSE16515, GSE32676, GSE1542, GSE74629, GSE91035, and GSE101462) (26–35). The clinical information of GSE62452 and GSE28735 was also downloaded to explore the potential prognostic value of SKA genes. After adding the TCGA PDAC cohort, a total of 715 PDAC samples and 297 non-cancer pancreatic samples from 12 datasets were combined and meta-analysis was used to compare gene expression between PDAC and normal tissue.

### Bioinformatic Analysis Using SKA Family Genes

The online tools of Gene Multiple Association Network Integration Algorithm (GeneMANIA)^5^ and the Search Tool for the Retrieval of Interacting Genes/Proteins (STRING)^6^ were used to structure interaction networks in gene–gene and protein–protein, respectively (36–39). A co-expression matrix of SKA mRNA was constructed using the TCGA, GTEx, and GSE62452 datasets.

### Survival Analysis

Kaplan–Meier (KM) survival analysis and multivariate Cox regression analysis were used to evaluate the SKA gene prognostic value. Samples in each dataset were divided into high and low expression groups based on each gene’s median expression value. Kaplan–Meier survival analysis was used to screen out clinical factors related to PDAC prognosis, and the clinical parameters with a *P* < 0.05 were selected into the Cox regression analysis. Next, multivariate survival analysis adjusted by prognostic-related clinical features was done. As the clinical information of GSE62452 and GSE28735 datasets was inadequate, these datasets were used to validate the prognostic value of each gene by KM survival analysis only. Afterward, to evaluate combined predictive potential on patient overall survival (OS), joint-effect survival analysis for the combination of mRNA transcriptional level and prognosis-related clinical factors was performed. Stratified survival analysis with multiple clinicopathological features was also used to evaluate the predictive value of the genes in PDAC patients.

### Nomogram

All PDAC patient data from TCGA and its matched clinicopathological features were combined with prognosis-related genes to construct a nomogram. Each prognosis-related gene was classified into the high or low expression groups based on median gene expression. The nomogram can calculate the total score of each patient based on existing information and predict survival probability. In addition, the nomogram can evaluate how each parameter affects the probability of survival.

### Prognostic Value Evaluation and Clinical Relevance of SKA1 and SKA3

Area under the curve (AUC) of the receiver operating characteristic (ROC) curve was done to assess the accuracy of prognostic-related genes in predicting patient survival using survivalROC package. We compared the correlation between expression of prognosis-related genes and clinicopathology features of PDAC using the datasets with adequate clinical information.

### Potential Mechanism and Regulatory Factors of Genes

Samples from TCGA were divided into two categories according to median expression values of prognosis-related SKA genes. Gene set enrichment analysis (GSEA)^7^ was then done to explore potential biological mechanisms (40, 41). Gene sets of Kyoto Encyclopedia of Genes and Genomes (KEGG) (c2.all.v6.2.symbols.gmt) and gene ontology (GO) (c5.all.v6.2.symbols.gmt) were used in this study (42, 43). The criteria for statistically significance were a nominal *P* value < 0.05 and FDR < 0.25. (44). Next, kinase–target, transcription factor–target, and miRNA network enrichment analyses were done on the LinkedOmics database^8^ online tool (45). The top five outcomes from each gene set are shown.

### Analysis of Genomic Alterations and Methylation Level of Prognosis-Related Genes

To elucidate the potential regulatory mechanism of gene expression, gene mutations, and copy number variations (CNVs), data were accessed on cBioportal.^9^ The calculation method of CNVs was Genomic Identification of Significant Targets in Cancer 2.0 (GISTIC2) (46). Next, DNA methylation data for the genes with matched RNA-seq expression profile (log2(count + 1)) were accessed from UCSC Xena and analyzed by Infinium Human Methylation 450 BeadChip. The methylation assessment for the genes was expressed as β-values. Correlation analysis and KM survival analysis were used to explore potential methylation sites regulating gene expression.

### Exploration of Tumor Immune Infiltration

RNA-seq data were analyzed using ESTIMATE (estimation of stromal and immune cells in malignant tumor tissues using expression data)^10^ algorithm to calculate cellular component in tumor tissues and estimate tumor microenvironment (TME) scores for stromal and immune cells (47). The immune cells analyzed to assess tumor immunity are B cells, CD4+ T cells, CD8+ T cells, dendritic cells (DCs), neutrophils, and macrophages. Tumor Immune Estimation Resource (TIMER)^11^ was then used to evaluate infiltration by the immune cells (48). Next, the correlation between immune cell infiltration and prognosis-related gene expression in PDAC was evaluated.

### Statistical Analysis

Stata (version 12.0) was used to plot forest plots and summary ROC (sROC) curve of the meta-analysis. The standard mean difference (SMD) and 95% confidence interval (CI) were used to identify differentially expressed SKA genes in cancer and non-cancer tissues. Summary ROC was used to evaluate the capacity of genes to discriminate between cancer and non-cancer tissues. All statistical analysis was carried out on SPSS (version 22.0). Survival differences were identified by hazard ratio (HR) and 95% CI. Two or multiple groups of continuous variables were compared by Student *t* test and one-way ANOVA, respectively. All the correlation analysis method used Spearman’s correlation. *P* value < 0.05 was considered statistically significant.

## Results

### Expression Level Distribution and Meta-Analysis of SKA1–3

**FIGURE 2:**
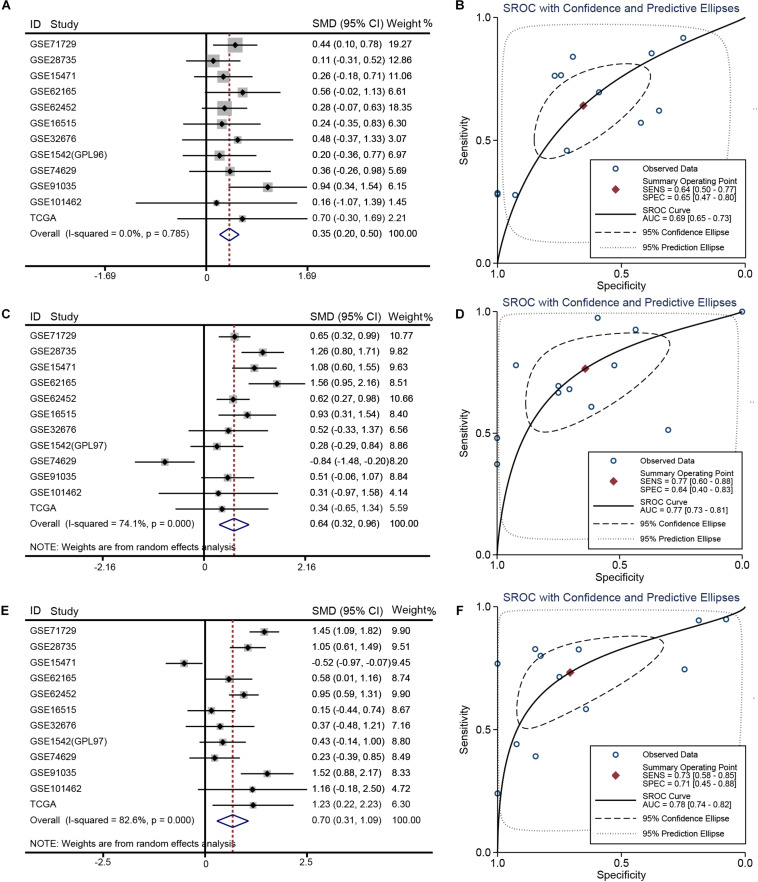
Meta-analysis of 12 datasets of pancreatic cancer. **(A)** Forest plot showing *SKA1* expression difference. **(B)** sROC curve of *SKA1*. **(C)** Forest plot showing *SKA2* expression difference. **(D)** sROC curve of *SKA2*. **(E)** Forest plot showing *SKA3* expression difference. **(F)** sROC curve of *SKA3*.

Relative to other human normal organ tissues, *SKA1*–*3* expression was lower in pancreatic tissues in GTEx (Supplementary Figures 2A–C). To obtain stability results of differential expression analysis of SKA genes between PDAC tissues and non-tumor tissues, we integrated multiple datasets for meta-analysis. This analysis showed that *SKA1*–*3* expression is higher in PDAC tissue relative to non-tumor pancreatic tissue [*SKA1*: SMD = 0.35 (0.20–0.50), *I*^2^ = 0.0%, Figure 2A; *SKA2*: SMD = 0.64 (0.32–0.96), *I*^2^ = 74.1%, Figure 2C; *SKA3*: SMD = 0.70 (0.31-1.09), *I*^2^ = 82.6%, Figure 2E]. The AUCs of the sROC of *SKA1*, *SKA2*, and *SKA3* as calculated by diagnostic meta-analysis were 0.69, 0.77, and 0.78, respectively (Figures 2B,D,F). Heterogeneity for meta-analysis of *SKA2* and *SKA3* was significantly derived from GSE74629 and GSE15471, respectively, whereas no heterogeneity existed in the comprehensive analysis of *SKA1*.

### Bioinformatics Analysis

**FIGURE 3:**
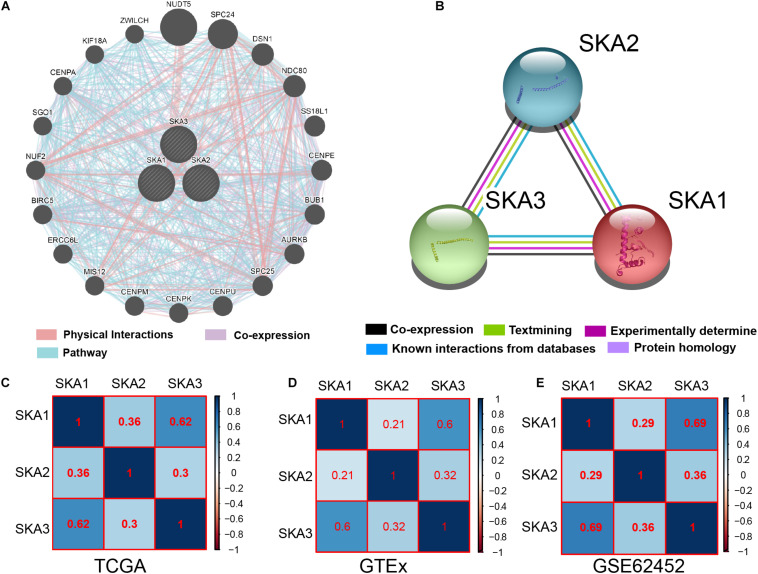
Interaction networks and co-expression matrix of spindle and kinetochore associated genes. **(A)** Gene–gene interaction network created using the Gene Multiple Association Network Integration Algorithm (GeneMANIA). **(B)** Protein–protein interaction network created using the Search Tool for the Retrieval of Interacting Genes/Proteins (STRING). **(C–E)** Co-expression matrix of SKA genes in TCGA, GTEx, and GSE62452 datasets.

Gene and protein interaction analysis *SKA1*–*3* using GeneMANIA and STRING, respectively, revealed a significant degree interaction, as well as protein homology (Figures 3A,B). Furthermore, there existed strong co-expression of SKA genes not only in the normal dataset of GTEx but also in tumor datasets of TCGA and GSE62452 (Figures 3C–E), especially the correlation between *SKA1* and *SKA3* (*r* = 0.62, 0.6, and 0.69).

### Survival Analysis

**FIGURE 4:**
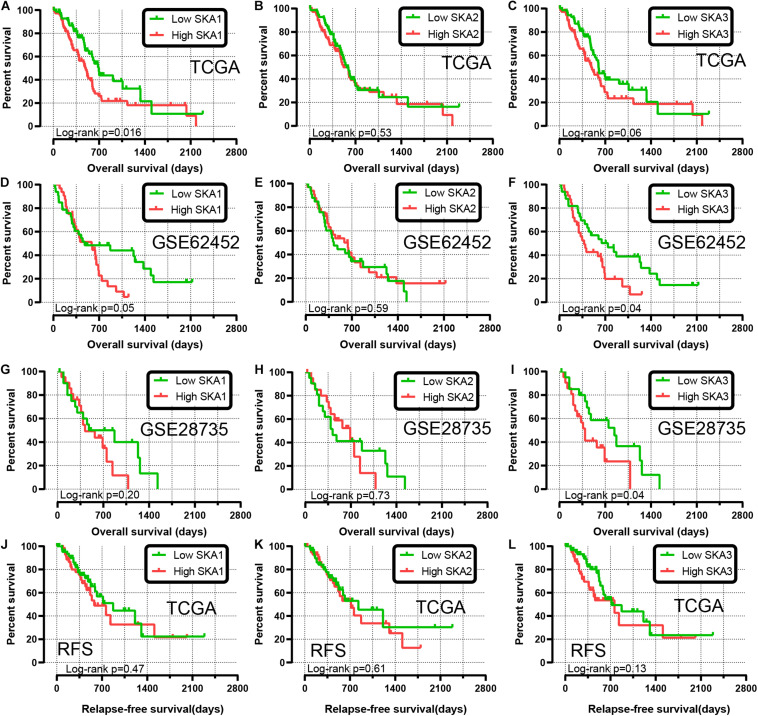
Kaplan-Meier survival curves showing the association of spindle and kinetochore related genes with the overall survival and relapse-free survival of pancreatic ductal adenocarcinoma patients from TCGA and GEO datasets. Overall survival in TCGA: **(A)** *SKA1*; **(B)** *SKA2*; **(C)** *SKA3*; Overall survival in GEO62452: **(D)** *SKA1*; **(E)** *SKA2*; **(F)** *SKA3*; Overall survival in GEO28735: **(G)** *SKA1*; **(H)** *SKA2*; **(I)** *SKA3*; Relapse-free survival in TCGA stratified by **(J)** *SKA1*; **(K)** *SKA2*; **(L)** *SKA3*.

We first analyzed TCGA dataset and removed a case lacking the survival time so that 145 eligible PDAC patients were enrolled at last. After performing KM survival analysis with the clinicopathological indicators of PDAC, the *P* value less than 0.05 clinical features including radical resection, radiation therapy, and targeted molecular therapy were selected out, which were further involved in the Cox regression analysis for adjustment (Table 1). Then, the KM method was also used for *SKA1–3* gene survival analysis, indicating that all the genes were significantly associated with prognosis in PDAC patients except for *SKA2*, and high expression suggest a shorter survival time (Figures 4A–C). The median survival time (MST) for low and high expression groups of SKA genes were *SKA1* (695 vs 485 days), *SKA2* (568 vs 518 days), and *SKA3* (698 vs 485 days) (Table 2). Similarly, the Cox regression analysis outcomes showed that *SKA1* (adjusted *P* = 0.04; adjusted HR = 1.656, 95% CI: 1.024–2.677) was an independent prognostic factor for PDAC patients as well as in *SKA3* (adjusted *P* = 0.034; adjusted HR = 1.688, 95% CI: 1.040–2.742), while it was not with *SKA2* (adjusted *P* = 0.837; adjusted HR = 0.952, 95% CI: 0.592–1.529). Survival analysis from GEO datasets of GSE62452 and GSE28735 showed that high expression of *SKA1* and *SKA3* was significantly correlated with a poor OS, except for *SKA1* in GSE28735 due to the limited sample (Figures 4D–I). To estimate the role of SKA genes in recurrence of PDAC, relapse-free survival (RFS) time was used to structure the KM curve, which suggests that the high expression group of *SKA1* or *SKA3* was not associated with the recurrence rate of PDAC compared with the low expression group. (Figures 4J–L).

**TABLE 1:** Clinical characteristics of pancreatic ductal adenocarcinoma patients.

Variables	Events/total (*n* = 145)	MST (days)	HR (95% CI)	Log-rank *P* value
**Age (years)**				0.406
<60	23/42	592	1	
≥60	60/103	568	1.405 (0.887,2.226)	
Missing	0			
**Gender**				0.412
Female	43/69	570	1	
Male	40/76	660	0.784 (0.55,1.118)	
Missing	0			
**TNM stage**				0.905
Stage I	6/12	498	1	
Stage II	74/125	592	1.608 (0.813,3.182)	
Stage III + IV	3/7	545	2.333 (1.069,5.090)	
Missing	1			
**Histologic grade**				0.164
G1/G2	56/104	607	1	
G3	27/41	485	1.366 (0.966,1.932)	
Missing	0			
**Radiation therapy**				0.023
Yes	58/94	481	1	
No	17/37	702	2.322 (1.418,3.802)	
Missing	14			
**Targeted molecular therapy**				< 0.001
Yes	27/35	239	1	
No	52/99	684	1.489 (1.055,2.1)	
Missing	11			
**Alcohol history**				0.923
No	29/50	532	1	
Yes	46/83	598	1.263 (0.794,2.007)	
Missing	12			
**Radical resection**				0.009
Yes	46/83	614	1	
No	33/53	394	1.691 (1.098,2.604)	
Missing	9			

*MST, median survival time; HR, hazard ratio; CI, confidence interval.*

**TABLE 2:** Prognostic value of single and combined of spindle and kinetochore-associated genes expression in pancreatic ductal adenocarcinoma patient OS from TCGA.

Gene	Events/total (*n* = 145)	MST/MRS (days)	Crude HR (95% CI)	Crude *P* value	Adjusted HR (95% CI)^a^	Adjusted *P* value^a^
***SKA1***						
Low	37/72	695	1		1	
High	46/73	485	1.711 (1.095,2.671)	0.018	1.656 (1.024,2.677)	0.04
***SKA2***						
Low	39/72	568	1		1	
High	44/73	518	1.149 (0.745,1.771)	0.530	0.952 (0.592,1.529)	0.837
*SKA3*						
Low	40/72	598	1		1	
High	52/73	485	1.495 (0.968,2.308)	0.069	1.688 (1.040,2.742)	0.034
**Group 1**						
*SKA1*^low^ + *SKA3*^low^	26/53	695	1		1	
**Group 2**						
*SKA1*^low^ + *SKA3*^high^ or *SKA1*^high^ + *SKA3*^low^	16/38	598	1.200 (0.641,2.246)	0.568	1.010 (0.482,2.115)	0.980
**Group 3**						
*SKA1*^high^ + *SKA3*^high^	41/54	394	1.797 (1.098,2.942)	0.02	1.587 (0.932,2.702)	0.089

*CI, confidence interval; SKA, spindle and kinetochore associated; HR, hazard ratio; MST, median survival time; OS, overall survival. ^*a*^Adjusted for radical resection, radiation therapy, and targeted molecular therapy.*

**FIGURE 5:**
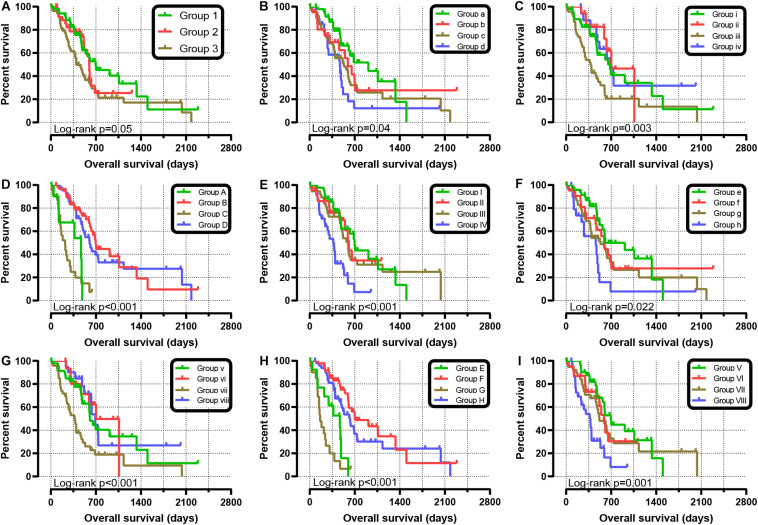
Joint-effect survival analysis for overall survival of patients with pancreatic ductal adenocarcinoma. **(A)** Combination of *SKA1* and *SKA3*. Combination of *SKA1* and prognosis-related clinical factors: **(B)** histologic grade; **(C)** radiation therapy; **(D)** targeted molecular therapy; **(E)** radical resection. Combination of *SKA3* and prognosis-related clinical factors: **(F)** histologic grade; **(G)** radiation therapy; **(H)** targeted molecular therapy; **(I)** radical resection.

We further explored the effect of the gene combination on the prognosis of patients and divided samples into three groups. KM survival curves showed that group 3 (MST: 394 days) with high expression of *SKA1* and *SKA3* genes had the worst prognosis, while group 1 (MST: 695 days) with low expression of the two genes had the best prognosis, suggesting that their high expression represents a high risk of death (Table 2 and Figure 5A). The combination of *SKA1* or *SKA3* with prognosis-related clinical characteristics can better show the difference in PDAC prognosis, which improved the predictive performance for prognosis (Figures 5B–I and Tables 3, 4).

**TABLE 3:** Joint-effect survival analysis of *SKA1* expression and clinical variables in pancreatic ductal adenocarcinoma patient overall survival.

Group	*SKA1*	Variables	Events/total (*n* = 145)	MST (days)	Crude HR (95% CI)	Crude *P* value	Adjusted HR (95% CI)^a^	Adjusted *P* value^a^
**Histologic grade**								
a	Low	G1 + G2	20/50	913	1		1	
b	Low	G3	12/22	592	1.413 (0.686,2.911)	0.349	2.030 (0.924,4.459)	0.078
c	High	G1 + G2	36/53	532	1.717 (0.991,2.975)	0.054	1.505 (0.828,2.734)	0.180
d	High	G3	16/20	470	2.459 (1.272,4.753)	0.007	1.759 (0.856,3.618)	0.125
**Radiation therapy^b^**								
i	Low	No	23/48	684	1		1	
ii	Low	Yes	7/18	702	0.718 (0.307,1.678)	0.444	1.002 (0.417,2.405)	0.997
iii	High	No	36/47	381	2.015 (1.192,3.407)	0.009	1.472 (0.848,2.554)	0.170
iv	High	Yes	10/18	691	0.839 (0.398,1.766)	0.644	0.817 (0.354,1.884)	0.636
**Targeted molecular therapy^c^**								
A	Low	No	7/11	467	1		1	
B	Low	Yes	25/57	695	0.239 (0.101,0.570)	0.001	0.287 (0.116,0.710)	0.007
C	High	No	20/24	219	1.512 (0.634,3.604)	0.351	1.659 (0.691,3.979)	0.257
D	High	Yes	28/42	603	0.290 (0.122,0.691)	0.005	0.329 (0.130,0.831)	0.019
**Radical resection^d^**								
I	Low	Yes	21/44	695	1		1	
II	Low	No	10/23	607	1.104 (0.518,2.354)	0.798	0.877 (0.384,2.006)	0.756
III	High	Yes	25/39	596	1.156 (0.641,2.084)	0.630	0.791 (0.425,1.469)	0.457
IV	High	No	24/31	366	2.949 (1.619,5.371)	< 0.001	2.440 (1.273,4.679)	0.007

**SKA1*, spindle and kinetochore-associated complex subunit 1; MST, median survival time; HR, hazard ratio; CI, confidence interval. ^*a*^Adjusted for radical resection, radiation therapy, targeted molecular therapy. ^*b*^Information of radiation therapy was unavailable in 14 patients. ^*c*^Information of targeted molecular therapy was unavailable in 11 patients. ^*d*^Information of radical resection was unavailable in 8 patients.*

**TABLE 4:** Joint-effect survival analysis of *SKA3* expression and clinical variables in pancreatic ductal adenocarcinoma patient overall survival.

**Group**	***SKA3***	**Variables**	**Events/total (*n* = 145)**	**MST (days)**	**Crude HR (95% CI)**	**Crude *P* value**	**Adjusted HR (95% CI)**^a^	**Adjusted *P* value**^a^
**Histologic grade^a^**								
e	Low	G1 + G2	21/48	607	1		1	
f	Low	G3	14/22	598	1.320 (0.667,2.611)	0.425	1.407 (0.666,2.974)	0.371
g	High	G1 + G2	35/55	532	1.614 (0.937,2.782)	0.085	1.731 (0.944,3.173)	0.076
h	High	G3	14/20	460	2.791 (1.412,5.517)	0.003	3.393 (1.605,7.172)	0.001
**Radiation therapy^b^**								
v	Low	No	24/48	607	1		1	
vi	Low	Yes	7/16	702	0.799 (0.343,1.861)	0.604	1.138 (0.448,2.891)	0.786
vii	High	No	35/47	375	2.268 (1.344,3.827)	0.002	2.094 (1.223,3.585)	0.007
viii	High	Yes	10/20	691	0.819 (0.391,1.715)	0.597	1.011 (0.451,2.270)	0.978
**Targeted molecular therapy^c^**								
E	Low	No	10/15	467	1		1	
F	Low	Yes	23/49	702	0.826 (0.412,1.654)	0.589	0.254 (0.112,0.574)	0.001
G	High	No	17/20	160	4.317 (2.069,9.008)	< 0.001	2.090 (0.942,4.638)	0.070
H	High	Yes	30/50	627	1.016 (0.517,1.995)	0.964	0.409 (0.182,0.917)	0.030
**Radical resection^d^**								
V	Low	Yes	21/43	695	1		1	
VI	Low	No	12/23	592	1.139 (0.578,2.242)	0.707	1.170 (0.527,2.598)	0.700
VII	High	Yes	25/40	517	1.347 (0.777,2.336)	0.289	0.718 (0.718,2.442)	0.369
VIII	High	No	22/31	378	3.427 (1.903,6.172)	< 0.001	1.702 (1.702,6.433)	<0.001

**SKA3*, spindle and kinetochore-associated complex subunit 3; MST, median survival time; HR, hazard ratio; CI, confidence interval. ^*a*^Adjusted for radical resection, radiation therapy, and targeted molecular therapy. ^*b*^Information of radiation therapy was unavailable in 14 patients. ^*c*^Information of targeted molecular therapy was unavailable in 11 patients. ^*d*^Information of radical resection was unavailable in 8 patients. New Pancreatic Cancer Prognostic Biomarkers New Pancreatic Cancer Prognostic Biomarkers.*

**FIGURE 6:**
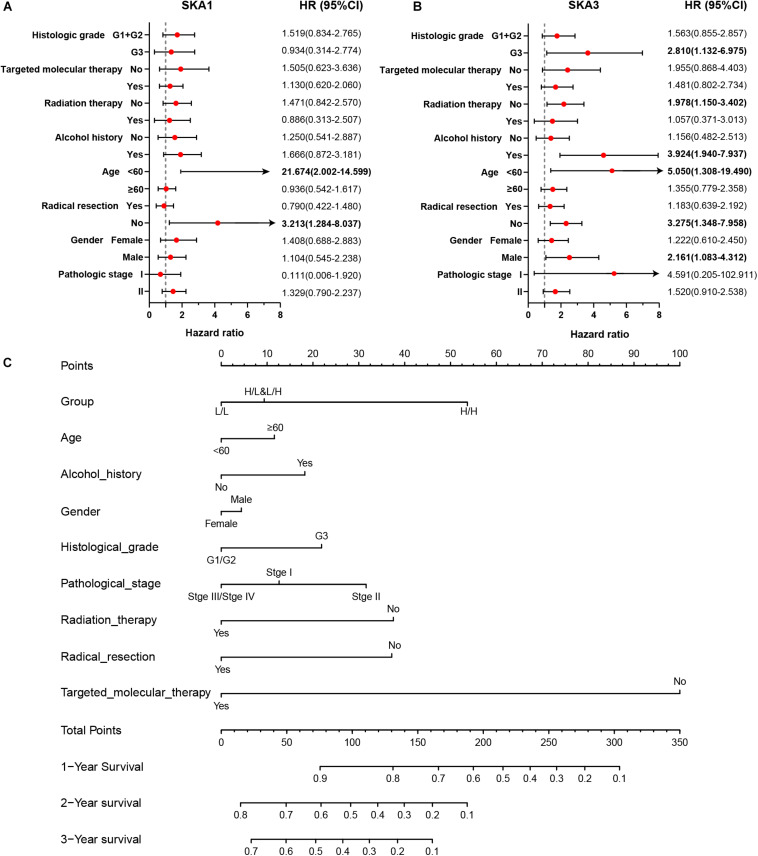
The relationship of spindle and kinetochore-associated genes with the clinical information. **(A,B)** Stratified survival analysis of *SKA1* and *SKA3* in various clinicopathological parameters. **(C)** A prognostic nomogram based on *SKA1* and *SKA3* for predicting the 1-, 2-, and 3-year overall survival rate of patients with pancreatic ductal adenocarcinoma. HR, hazard ratio; CI, confidence interval; L/L: *SKA1^*low*^* + *SKA3*^*low*^; L/H*: SKA1^*low*^* + *SKA3*^*high*^; H/L: *SKA1^*high*^* + *SKA3*^*low*^; H/H: *SKA1^*high*^* + *SKA3*^*high*^.

Stratified analysis with various clinical pathological parameters was adjusted by targeted molecular therapy, radiation therapy, and residual resection. High expression of *SKA1* suggested a poor survival time in patients with an age of <60 [HR (95% CI): 21.674 (2.002–14.599)] and non-radical resection [HR (95% CI): 3.213 (1.284–8.037)], and similarly, poor prognosis was significantly correlated with high expression of *SKA3* in patients with G3 [HR (95% CI): 2.810 (1.132–6.975)], no radiation therapy [HR (95% CI): 1.978 (1.150–3.402)], alcohol history [HR (95% CI): 3.924 (1.940–7.937)], age of < 60 [HR (95% CI): 5.050 (1.308–19.490)], non-radical resection [HR (95% CI): 3.275(1.384–7.958)], and male [HR (95% CI): 2.161 (1.083–4.321)] (Figures 6A,B).

### Nomogram

Patients were grouped into three categories as mentioned previously, and patient mRNA expression data with matched clinical features were used to build a nomogram. Each variable had a score that was denoted by a line length. The higher the score, the greater the effect of the gene on prognosis. This analysis reveals that that mRNA expression of *SKA1* and *SKA3* can significantly affect patient survival (Figure 6C).

### Prognostic Value and Clinical Relevance of Prognosis-Related Genes

**FIGURE 7:**
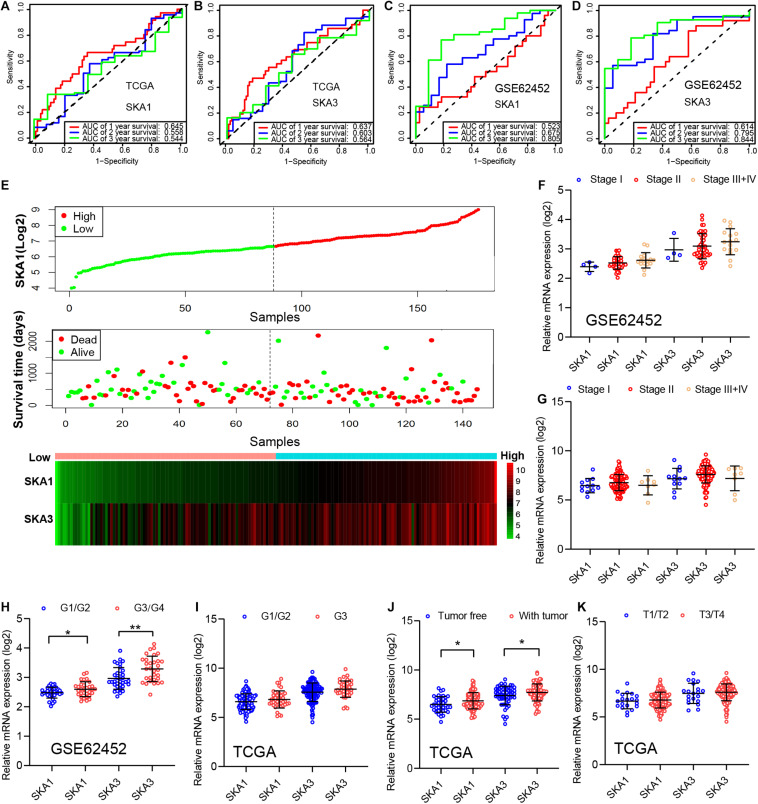
Analysis of the prognostic value and clinical relevance of *SKA1* and *SKA3* in pancreatic ductal adenocarcinoma patients. **(A–D)** Time-dependent receiver operating characteristic (ROC) curves of *SKA1* and *SKA3* showing the 1-, 2-, and 3-year overall survival rate of patients with PDAC from TCGA and GSE62452 datasets. **(E)** From top to bottom are the expression values of *SKA1*, patients’ survival status distribution, and the expression heat map of *SKA1* in the low- and high-expression groups. The expression distribution of *SKA1* and *SKA2* genes in different **(F,G)** AJCC stages and **(J,K)** grades in TCGA and GSE62452 datasets. **(H,I)** The expression distribution of *SKA1* and *SKA2* genes in different pathological T grade and cancer status in TCGA cohort. *p < 0.05, **p < 0.01, ***p < 0.001.

In TCGA cohort, the time-dependent ROC curve of *SKA1* (1, 2, and 3 years: 0.645, 0.558, and 0.544) and *SKA3* (1, 2, and 3 years: 0.637, 0.603, and 0.564) at 1-, 2-, and 3-year survival were medium as shown in Figures 7A,B. The time-dependent ROC curve produced from the GSE62452 dataset showed that *SKA1* (1, 2, and 3 years: 0.523, 0.675, and 0.805) and *SKA3* (1, 2, and 3 years: 0.614, 0.795, and 0.844) were effective predictors of 3-year PDAC survival (Figures 7C,D). Next, *SKA1* and *SKA3* expression scatter plots, survival status scatter diagrams, and expression heat maps were used to visualize the genes’ prognostic value. This analysis revealed that increased SKA expression levels correlated with reduced survival time (Figure 7E).

Subsequently, we evaluated correlation between prognosis-related genes and clinicopathological parameters. Analysis of the GSE62452 dataset revealed *SKA1* upregulation in stage II patients relative to stage I patients (Figures 7F,G). In terms of histologic grade, analysis of the GSE62452 dataset showed that *SKA1* and *SKA3* expression were significantly higher in G3/G4 PDAC relative to G1/G2 disease stage and *SKA1* and *SKA3* only showed an upregulated trend in G3 samples in TCGA (Figures 7H,I). Moreover, relative to tumor-free survival patients, tumor-bearing patients significantly expressed high levels of *SKA1* and *SKA3* (Figure 7J) and both of them did not corelate with advanced pathological T (T3/4) (Figure 7K).

### Gene Enrichment Analysis

**FIGURE 8:**
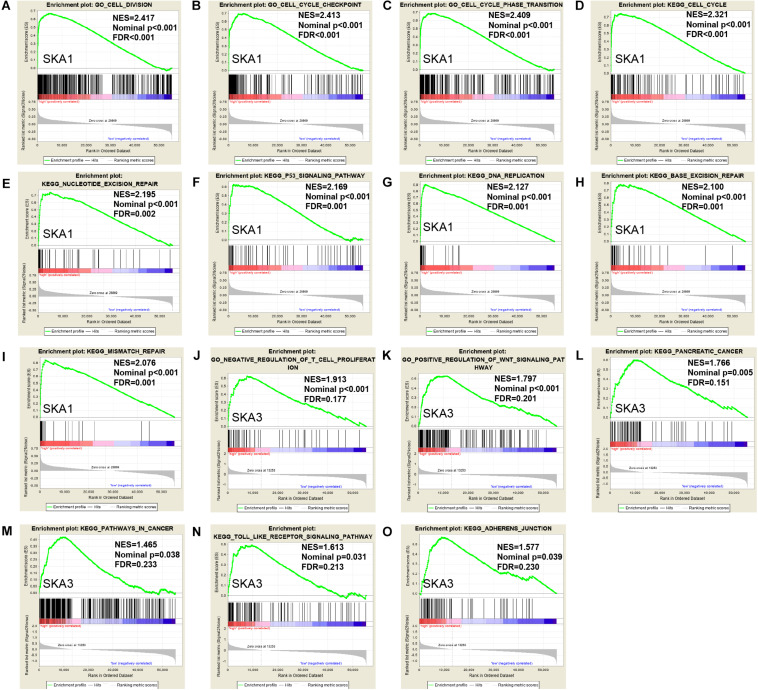
Gene set enrichment analysis of *SKA1* and *SKA3* in TCGA dataset. **(A–C, J, K)** GSEA results of C5 gene sets for high *SKA1* and *SKA3* expression groups; **(D–I, L–O)** GSEA results of C2 gene sets for high *SKA1* and *SKA3* expression groups; NES, normalized enrichment score; FDR, false discovery rate.

Gene set enrichment analysis analysis of the potential mechanism by which prognosis-related genes influence PDAC revealed that in the high *SKA1* expression group, there was significant enrichment for cell cycle-related biological processes (GO: cell division, cell cycle checkpoint, and cell cycle phase transition, Figures 8A–C) and tumor-related signaling pathways (KEGG: cell cycle, P53 signaling pathway, and DNA replication, Figures 8D–I). GO term analysis of the high *SKA3* expression group revealed enrichment for processes involved in negative regulation of T cell proliferation and positive regulation of the WNT pathway (Figures 8J,K). KEGG annotation showed the high *SKA3* expression group participated in tumor-related signaling pathways (pancreatic cancer, pathways in cancer, toll-like receptor pathway, and adherens junction) (Figures 8L–O). The enrichment results of network analyses on the LinkedOmics database indicated that SKA1 and SKA3 are mainly regulated by the same transcription factors (V$E2F1_Q6, V$E2F_Q4, V$E2F_Q6, V$E2F_Q4_01), networks, and kinases, including cyclin-dependent kinase 1 (*CDK1*), polo-like kinase 1 (*PLK1*), cyclin-dependent kinase 2 (*CDK2*), and aurora B kinase (*AURKB*). The miRNA-target network for *SKA1* was related to MIR-185, MIR-512-3p, MIR-507, MIR-218, and MIR-96, while *SKA3* was associated with MIR-507, MIR-119A, MIR-513, MIR-338, and MIR-137 (Table 5).

**TABLE 5:** The kinase and transcription factor-target networks of *SKA1* and *SKA3* in TCGA dataset (LinkedOmics).

Gene	Enriched Category	Gene Set	Leading-EdgeNum	FDR
*SKA1*	Kinase Target	Kinase_CDK1	69	0
		Kinase_PLK1	30	0
		Kinase_CDK2	58	0
		Kinase_AURKB	25	0
		Kinase_CHEK1	23	0
	Transcription Factor Target	V$E2F1_Q6	58	0
		V$E2F_Q4	47	0
		V$E2F_Q6	47	0
		V$E2F_Q4_01	45	0
		V$E2F_02	50	0
	miRNA Target	TCTCTCC,MIR-185	54	0
		CAGCACT,MIR-512-3P	66	0
		GTGCAAA,MIR-507	42	0
		AAGCACA,MIR-218	136	0
		GTGCCAA,MIR-96	99	0
*SKA3*	Kinase Target	Kinase_CDK1	66	0
		Kinase_AURKB	31	0
		Kinase_PLK1	30	0
		Kinase_CDK2	67	0
		Kinase_ATR	15	0
	Transcription Factor Target	V$E2F_Q3_01	44	0
		V$E2F1_Q4_01	43	0
		V$E2F_Q4_01	44	0
		V$E2F1_Q6	58	0
		V$E2F_Q6	45	0
	miRNA Target	GTGCAAA,MIR-507	52	0
		CTACTGT,MIR-199A	42	0.007
		CCTGTGA,MIR-513	37	0.009
		ATGCTGG,MIR-338	28	0.013
		AAGCAAT,MIR-137	59	0.013

*LeadingEdgeNum, the number of leading edge genes; FDR, false discovery rate from Benjamini and Hochberg from gene set enrichment analysis (GSEA). V$, the annotation found in Molecular Signatures Database (MSigDB) for transcription factors (TF).*

### Genomic Alterations and DNA Methylation Level of Prognosis-Related Genes

**FIGURE 9:**
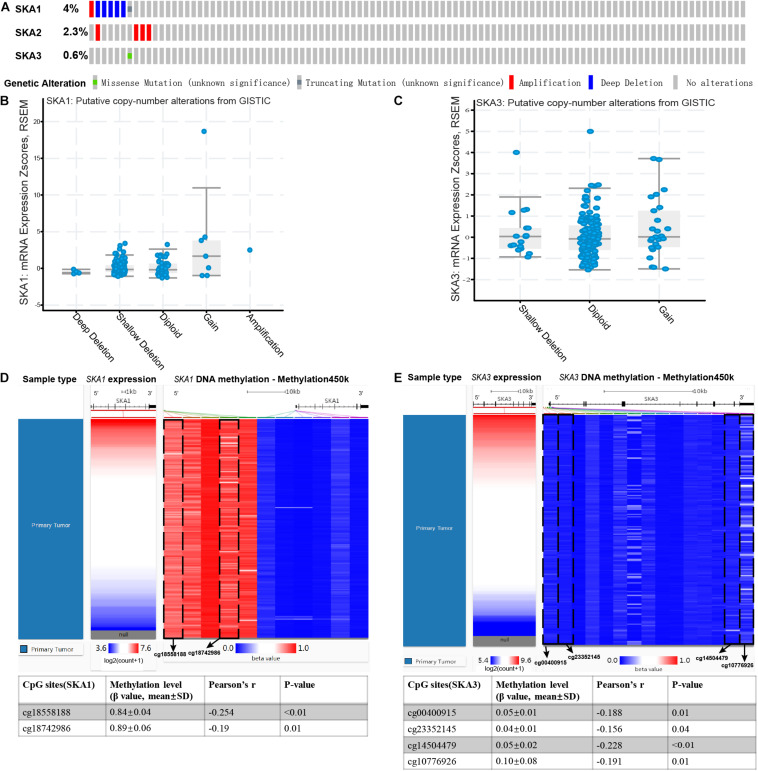
Genomic alterations and DNA methylation level of spindle and kinetochore-associated genes. **(A)** OncoPrint of *SKA1–3* alterations in TCGA cohort. Different types of genetic alterations shown in different colors. **(B,C)** *SKA1* and *SKA3* expression in different CNV groups. Data were obtained from the cBioportal (https://www.cbioportal.org/). **(D,E)** Two heatmaps showing the methylation profile of 11 CpG sites in *SKA1* DNA locus and 15 CpG sites in *SKA3* DNA locus. Data were obtained from UCSC Xena (http://xena.ucsc.edu/) using Infinium HumanMethylation450 BeadChip.

Analysis of SKA gene mutation in the 153 PDAC patients showed that 12 (7%) of them were mutation carriers (Figure 9A). Furthermore, box plot analysis of CNV data from the PDAC patients revealed that increased copy number of the genes poorly correlates with higher expression of prognosis-related genes (Figures 9B,C). First, We found poor correlation between DNA methylation and gene expression in pure PDAC (data not show). Next, we explored whether DNA methylation status influenced gene expression in 185 pancreatic cancer samples by analyzing 11 and 15 CpG sites in the *SKA1* and *SKA3* DNA locus, respectively. Five of the 11 sites in the *SKA1* DNA locus had high methylation with β values > 0.6, while two sites (cg18558188: β ± SD = 0.84 ± 0.04, | *r*| = 0.254; cg18742986: β ± SD = 0.89 ± 0.06, | *r*| = 0.19) exhibited significant negative correlation with gene expression, indicating that DNA methylation may regulate *SKA1* expression (Figure 9D). When samples were divided into two groups based on median methylation level, loci cg18558188 and cg18742986 methylation were not associated with PDAC OS, while higher total CpG methylation levels of *SKA1* correlated with improved patient survival (Supplementary Figures 3A–C). All *SKA3* CpG sites exhibited very low methylation (β-value < 0.4) and were not explored further (Figure 9E).

### Prognosis-Related Genes Correlated With Tumor Immune Microenvironment and Key Gene Mutations

**FIGURE 10:**
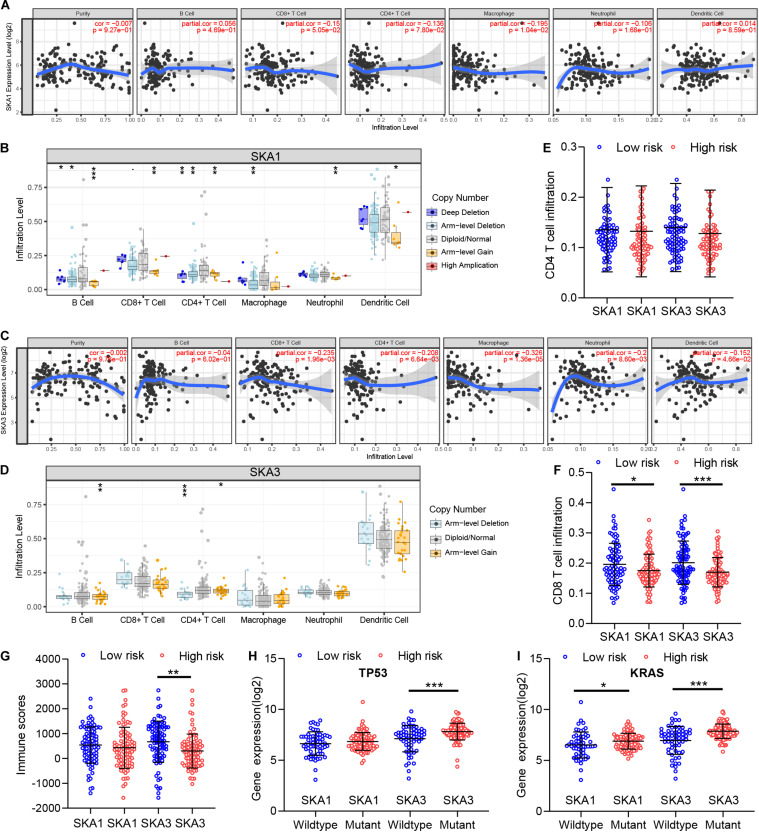
The impact of *SKA1* and *SKA3* gene expression and mutation on tumor immunity. **(A)** *SKA1* expression showed significant negative correlation with infiltration levels of CD8+ T cells and macrophages. **(B)** *SKA1* CNV influenced the infiltration levels of all the immune cells. **(C)** *SKA3* expression showed significant negative correlation with infiltration levels of CD8+ T cells, CD4+ T cells, macrophages, neutrophils, and dendritic cells. **(D)** Changes in *SKA3* CNV altered infiltration levels of B cells and CD4+ T cells. **(E–G)** Infiltration level of CD4+ T and CD8+ T cell and distribution of immune scores in high expression and low expression groups of *SKA1* and *SK A3* in TCGA datasets. Immune scores were calculated using the ESTIMATE algorithm. **(H,I)** The expression distribution of *SKA1* and *SKA3* genes in different mutation status of TP53 and KRAS in TCGA dataset. *p < 0.05, **p < 0.01, ***p < 0.001.

GSEA revealed that *SKA3* modulates T cell activity. Next, we explored relationship between prognosis-related genes and tumor immunity, including immune cell infiltration and immune scores. Results from TIMER analysis show that CD8+ T cells, CD4+ T cells, and macrophages exhibit a significant negative correlation with *SKA1* mRNA expression and its CNVs significantly impact immune cell infiltration levels (Figures 10A,B). In particular, *SKA3* expression significantly exhibits a strongly negative correlation with all immune cell infiltration levels except for B cell. Infiltration of natural killer (NK) cells is not related to SKA gene expression (Supplementary Figures 4A–D). Additionally, infiltration levels for B cell and CD4+ T cell can be affected by *SKA3* CNVs (Figures 10C,D). Then, scatter plot analysis was used to visualize the distribution of CD4+ T and CD8+ T cell between the groups expressing high and low levels of prognosis-related genes. This analysis revealed that CD8+ T cells were significantly fewer in the high expression group, but no difference in CD4+ T cell distribution was observed between the groups (Figures 10E,F). So, we speculated that high expression levels of *SKA1* and *SKA3*, to some extent, may mediate tumor escape and inhibit the infiltration levels of immune cells. To test this possibility, we calculated the tumor immune scores using the RNA-seq data from the TCGA cohort. Consistently, immune score negatively correlated with *SKA3* expression but did not correlate with *SKA1* expression, indicating that *SKA3* has a great influence on immune cell infiltration in PDAC tissues (Figure 10G). Tumor cells have the capacity to evade clearance by macrophages through the upregulation of antiphagocytic surface proteins including cluster of differentiation 47 (*CD47*), programed death-ligand 1 (*PD-L1*), programed cell death protein 1 (*PD1*), cytotoxic T-lymphocyte-associated protein 4 (*CTLA4*), and beta-2-microglobulin (*B2M*) (49–52). We explored whether *SKA1* and *SKA3* had correlation with these antiphagocytic molecules. *SKA1* and *SKA3* were positively correlated with the expression of *CD47*, *PD-L1*, and *B2M* in TCGA dataset, respectively. Similar results were displayed in the GSE625452 dataset (Supplementary Table 2). These results may explain why high expression of *SKA1* and *SKA3* could result in reduced infiltration levels of immune cells in PDAC. Mutation analysis revealed that high *SKA1* expression highly correlates with the KRAS mutation group but not with TP53 mutations. High *SKA3* expression in the KRAS and TP53 mutant groups was significantly associated with the mutant type (Figures 10H,I).

## Discussion

In this study, we combined PDAC cohorts’ data from GEO and TCGA and used bioinformatics to evaluate the potential prognostic value of SKA genes in PDAC. Our meta-analysis found that all SKA genes are significantly upregulated in PDAC and exhibited medium diagnostic value for PDAC. Survival analysis showed that overexpression of *SKA1* and *SKA3* is associated with poor prognosis. The prognostic value of the two genes was further studied by ROC, joint-effect, and stratified analysis among other strategies. Our data highlight *SKA1* and *SKA3* as potential therapeutic targets against PDAC. Additionally, we explored the potential mechanisms regulating *SKA1* and *SKA3* expression and found that DNA methylation may influence *SKA1* expression and patients’ OS. Possible mechanisms include cancer- and immune-related pathways, and potential regulators include cancer-related kinases, miRNA, and the E2F family. Finally, we explored the association between prognosis-related genes and tumor immune microenvironment.

Biochemically, *SKA1* is known to directly bind microtubules through its C-terminal domain to stimulate oligomerization (11, 53). Spindle and kinetochore-associated complex deficiency is associated with chromosome congression failure and cell death (54–56). Multiple studies have reported that *SKA1* promotes cancer progression in a variety of tumors (13, 19, 57–61). An *in vitro* study found that *SKA1* accelerates cell proliferation and cancer progression in glioma via tumor-associated signaling pathways, including cell cycle and Wnt/β-catenin signaling (62). An immunohistochemical study of 126 hepatocellular carcinoma patients revealed that *SKA1* expression is significantly elevated in tumor tissues, and it can regulate the hepatocellular carcinoma cell cycle and contributes to poor prognosis (63). Qin et al. used targeted small interfering RNA to knockdown *SKA1* and observed hepatocellular carcinoma cell cycle arrest in the G0/G1 phase (17). Besides, *SKA1* has been reported to contribute to chemotherapy resistance in lung carcinoma through prevention of cisplatin-induced apoptosis (19). Here, we find that high *SKA1* expression predicts poor PDAC prognosis and participates in cell cycle, cell cycle checkpoint, P53 signaling pathway, and DNA replication, processes that significantly correlate with cancer progression (64–67). Our data also associated high *SKA1* expression with advanced cancer phenotypes such as stage II, G3 + G4, and patients survived with tumor. *SKA2*, a novel cell cycle gene, has been proposed as a biomarker and therapeutic target against cancer (68). It has been reported that suppressed *SKA2* expression triggers kinetochore fiber instability, leading to mitotic failure (9). A study by Ren et al. found that *SKA2* overexpression induces epithelial–mesenchymal transition in breast cancer, promoting cell invasion and metastasis (69). *SKA2* also influences proliferation, migration, and invasive capacity of gastric and lung cancer cells (70, 71). While *SKA2* was also highly expressed in our study, no correlation was found with PDAC prognosis. A probable reason for this may be inter-tumor heterogeneity, which requires additional investigation. *SKA3* mediates appropriate mitotic exit by interacting with the NDC80 complex, which regulates meiotic spindle migration and anaphase spindle stability (55, 72, 73). *SKA3* upregulation in lung adenocarcinoma cells correlates with increased metastases and tumor growth (74). Similarly, it has been reported that high *SKA3* expression promotes lung cancer cell proliferation and predicts patient outcomes. Through bioinformatics analysis, Tang et al. found that *SKA3* is associated with elevated susceptibility to breast cancer brain metastasis and negatively correlates with breast cancer survival (75). Our data further validate the correlation between high *SKA3* expression and advanced clinical features of G3/G4. Our findings show that *SKA3* may be involved in Wnt signaling, pancreatic cancer, pathways in cancer, toll-like receptor signaling pathway, and adherens junction, which are known to play a crucial role in tumor progression (76–78).

Kinases regulate various processes such as genomic stability, mitosis, and the cell cycle. *CDK1*, a member of the cyclin-dependent kinase protein family, participates in mitosis, cell cycle, cell differentiation, and somatic reprograming (79). Previous investigators have shown that dysregulation of *CDK1* leads to G2 phase arrest and promotes tumor progression, making it an ideal biomarker and therapeutic target (80, 81). *CDK1* binds Ndc80, thereby phosphorylating *SKA3* and recruiting SKA to kinetochores to facilitate mitotic progression (82). Another study by Hou et al. showed that *SKA3* interacted with *CDK2* and inhibited P53 phosphorylation, thereby regulating proliferation of liver cancer cell (83). *PLK1*, a tumorigenic factor, is a mitotic cyclin-independent serine threonine kinase that has been proposed as a potential therapeutic target for pancreatic cancer (84, 85). Another kinase, *AURKA*, participates in pancreatic carcinogenesis via the MAPK1/ERK2 signaling pathway (86). The same regulatory network of kinases (*CDK1*, *CDK2*, *PLK1*, and *AURKA*) was identified in our study as potential regulators of *SKA1* and *SKA3* in PDAC. The E2F family is part of the transcription factors that regulate gene expression. It participates in the cell cycle process, and activated E2F initiates oncogenic signaling in several cancer types (87, 88). Several approaches have been developed to directly or indirectly target *E2F1* with the aim of modifying malignant phenotypes of PDAC (89–92). Herein, *E2F1* was found to work with *SKA1* and *SKA3* to jointly regulate cell cycle and aggravate PDAC. MicroRNA, an endogenous small RNA with a length of about 20–24 nucleotides, has been implicated in human carcinogenesis (93). The miRNAs identified in our study have been associated with deteriorated neoplastic malignant phenotype such as proliferation, cell cycle, invasion, drug resistance, and angiogenesis (94–98). In fact, miR-185, miR-96, miR-218, miR-137, and miR-338 have been proven to exhibit therapeutic and prognostic value in PDAC, indicating that the SKA gene may be one of the target genes for these miRNAs (99–103).

A research team found that genomic alteration is a common phenomenon in human tumors (104). Copy number variations, induced by genomic rearrangement, disrupt genes and alter genetic content, causing different phenotypes. In this study, CNVs of *SKA1* and *SKA3* were found to be weakly correlated with gene expression. DNA methylation is a crucial epigenetic mechanism, which maintains genome stability, chromatin structure, and pluripotency in human cells. Changes in DNA methylation levels often accompany neoplasm development (105, 106). The role of DNA methylation in SKA genes has not been investigated. In this preliminary study, we found that two sites in *SKA1* were significantly involved with gene expression. High total DNA methylation level of *SKA1* predicts favorable prognosis in PDAC. We thus hypothesize that DNA methylation may be an important regulatory mechanism for *SKA1* that influences the OS of patients with PDAC. However, no DNA methylation sites were found to regulate *SKA3* gene expression, which may need more work to test it.

In recent years, immunotherapy has shown great promise for cancer patients, especially those with refractory cancer. The complexity of immune cells in tumor tissue influences the host biological behavior and the outcome of immunotherapy (107). The TME, composed of non-cancerous cells in and around the tumor, also performed an important role in the genomic analysis of various tumors (108). CD8+ cytotoxic T lymphocytes (CTLs) can specifically recognize major histocompatibility complex (MHC) antigens, which are widely used in targeted therapy (109). A pioneer study revealed that infiltration of CD4+/CD8+ T cells correlated with good prognosis in PDAC (110). Likewise, a study by Masugi et al. (111) measured densities of CD8+ T cells in different locations of tumor. They found that high CD8+ T cell density was significantly associated with prolonged survival of 214 patients with pancreatic cancer (111). Wu et al. also reported that the low infiltration level of CD4+ T lymphocytes was associated with poor prognosis of pancreatic cancer patients (112). GSEA suggested that *SKA3* negatively regulated T cells. Thus, we assessed the association between immune cell infiltration and SKA prognosis-related genes in PDAC. Results showed that *SKA1* was negatively correlated with CD8+ T cell and macrophages, and the infiltration level of CD8+ T cells was significantly lower in the high *SKA1* expression group, indicating an immunosuppressive state. Similarly, the abundance of CD4+ T cells, CD8+ T cells, and macrophages was decreased in the *SKA3* high expression group. There is a widespread belief that CD4+ T cells compromise the majority of T cells in pancreatic cancer and are positively associated with tumor metastasis and negatively associated with OS (113). Some subsets of CD4+ T cells may also be needed for antitumor immunity. CD4+ helper T cells may promote and maintain CTL memory, amplify T and B cells, and help CTL resist negative regulation (114). CD4+ T lymphocytes may inhibit tumor cell growth by cytolysis or by regulating the TME (115). More detailed studies are necessary to illustrate the specific role of each CD4+ T lymphocyte subset in pancreatic cancer. Macrophages, participating in the production, mobilization, activation, and regulation of immune effector cells, have at least three major functions: antigen presentation, phagocytosis, and immunomodulation (116). Cytokines such as IL-1, IL-6, TNF-α, interferon (IFN)-α/β, IL-10, IL-12, and IL-18 released from macrophages could participate in the regulation of immune/inflammatory responses. IL-12 stimulates proliferation of activated T and NK cells, enhances NK and lytic activity of CTLs, and induces IFN-γ production by T and NK cells. In addition, they produce chemokines that stimulate lymphocyte movement and regulation of migration lymphocytes from the blood to tissues (117). Similarly to macrophages, DCs have long been established as indispensable antigen-presenting cells (APCs), which act as systemic sentinels capable of responding to endogenous and exogenous “danger” signals to initiate and propagate immune responses to inciting antigens or induce immune tolerance (118, 119). On sensing of appropriate cues, DCs mature and express chemokine receptors and costimulatory molecules under normal conditions. DCs promote immunity or tolerance by sampling and presenting antigens to T cells and by providing immunomodulatory signals through cell–cell contacts and cytokines (120, 121). DCs are often associated with superior cross-presentation of antigens, which results in stronger CD8+ T cell immunity, and DCs can additionally support T helper 1 cell polarization of CD4+ T cells (122, 123). In tumor patients, DCs acquire, process, and present tumor-associated antigens on MHC molecules and provide costimulation and soluble factors to shape T cell responses. However, a number of active mechanisms in the TME perturb DC functions, resulting in insufficient T cell activation and, potentially, the induction of T cell tolerance to tumor-associated antigens (119). Lymphocytes and macrophages decreased significantly in patients with high expression of *SKA1* and/or *SKA3* in this study, leading to reduction of activation of immune cells, including CD8+ T and CD4 + T cells and NK and lytic activity of CTLs, suggesting that they might have an immune-excluded phenotype where CD4+ T/CD8+ T cells were maintained in the stroma, restricting cancer immunity. Interestingly, the infiltration of cytotoxic CD8+ T cells might be modified by immunotherapy. The above mechanism may be potentially responsible for short-term survival in PDAC patients correlates with increased *SKA1* and/or *SKA3* expresses. Analysis of immune scores in TME yielded similar outcomes as those of immune infiltration. In addition, *SKA1* and *SKA3* were positively correlated with the expression of antiphagocytic-related genes (*CD47*, *PD-L1*, and *B2M*). In general, these findings indicate that *SKA1* and *SKA3* play a significant role in the recruitment and regulation of immune-infiltrating cells in PDAC, which may eventually influence patients’ survival time. Thus, we hypothesized that patients with high expression of *SKA1* or *SKA3* might benefit from immunotherapy than those with low expression. Further research is required to address this hypothesis.

Finally, some limitations exist in this study. First, this study was performed using retrospectively collected data, which may contain selection bias and recall bias. Secondly, our results are based on bioinformatics analysis and thus underlying biological mechanisms remain undefined. Thirdly, the protein expression levels of *SKA1* and *SKA3* and their involvement in the pathogenesis and progression of PDAC deserve further studies. Despite these limitations, this is the first study to reveal the potential correlation between SKA genes and PDAC tumor immune escape and comprehensively explore the prognostic value of SKA genes in patients with pancreatic cancer. Our results may be informative for future research and clinical management of PDAC patients. Despite these limitations, this is the first study to reveal the association of SKA genes with immune function regulation of PDAC patients. There have been no reports demonstrating that SKA genes could regulate the immune infiltration of PDAC.

Collectively, high expression of *SKA1* and *SKA3* predicts poor prognosis of PDAC and may therefore be potential biomarkers for this disease. These genes could regulate cancer-related signaling pathways and inhibit immune infiltration within the tumor in PDAC. Further prospective studies are required to verify these molecular mechanisms.

## Data Availability Statement

The datasets presented in this study can be found in online repositories. The names of the repository/repositories and accession number(s) can be found in the article/ Supplementary Material.

## Ethics Statement

Ethical review and approval was not required for the study on human participants in accordance with the local legislation and institutional requirements. Written informed consent for participation was not required for this study in accordance with the national legislation and the institutional requirements.

## Author Contributions

XH, YC, and YL performed the data analysis work and aided in writing the manuscript. QL and ZJ designed the study and assisted in writing the manuscript. YL and QL edited the manuscript. All authors read and approved the final manuscript.

## Conflict of Interest

The authors declare that the research was conducted in the absence of any commercial or financial relationships that could be construed as a potential conflict of interest.

## Footnotes

1 https://www.gtexportal.org/home/

2 http://cancergenome.nih.gov

3 http://xena.ucsc.edu/

4 https://www.ncbi.nlm.nih.gov/geo/

5 http://genemania.org/

6 https://string-db.org/cgi/input.pl

7 http://www.broadinstitute.org/gsea/index.jsp

8 http://www.linkedomics.org/login.php

9 https://www.cbioportal.org/

10 https://bioinformatics.mdanderson.org/public-software/estimate/

11 https://cistrome.shinyapps.io/timer/

## Supplementary Material

Supplementary Figure 1 A flow diagram showing the selection process of GEO datasets.

Supplementary Figure 2 The distribution of **(A)** *SKA1*, **(B)** *SKA2*, **(C)** *SKA3* genes in human normal organ tissues.

Supplementary Figure 3 Kaplan-Meier survival curves showing the survival significance of *SKA1* DNA methylation in pancreatic cancer patients. **(A)** Total methylation level of *SKA1*; **(B)** cg18558188; **(C)** cg18742986.

Supplementary Figure 4 The impact of *SKA1* and *SKA3* gene expression and mutation on tumour immunity. **(A,B)** *SKA1* expression showed no correlation with infiltration levels of natural killer (NK) cells. **(C,D)** *SKA3* expression is not associated with infiltration levels of natural killer (NK) cells.

Supplementary Table 1 Details of the GEO datasets included in this study.

Supplementary Table 2 Correlations between prognosis-related gene and Gene Markers of cancer escape in TCGA and GSE62452.
