# Supplementary figures and images for: Identification of Spindle and Kinetochore-Associated Family Genes as Therapeutic Targets and Prognostic Biomarkers in Pancreas Ductal Adenocarcinoma Microenvironment
Source: Front Oncol. 2020 Nov 2;10:553536. doi: 10.3389/fonc.2020.553536 (PMC7667267; doi:10.3389/fonc.2020.553536)

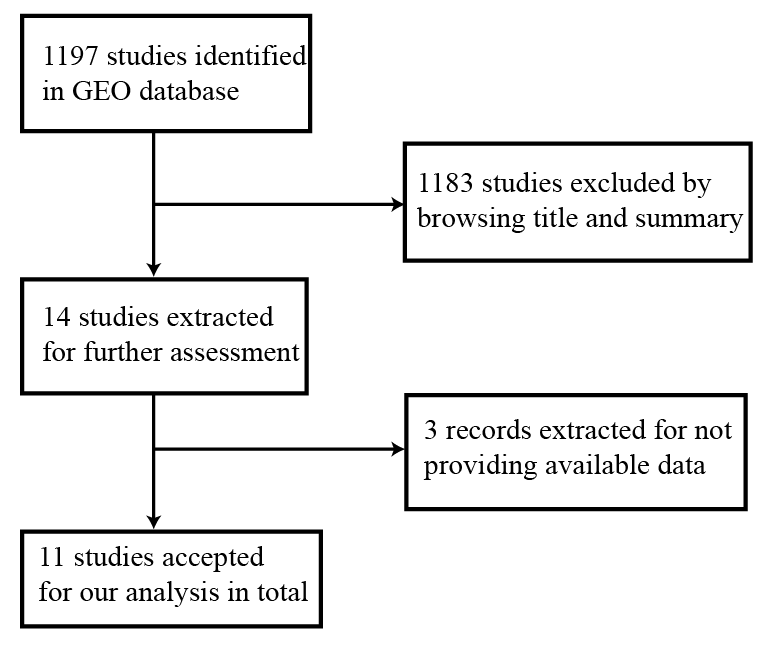

Supplement: Supplementary Figure 1 — A flow diagram showing the selection process of GEO datasets. [file Image_1.TIF]

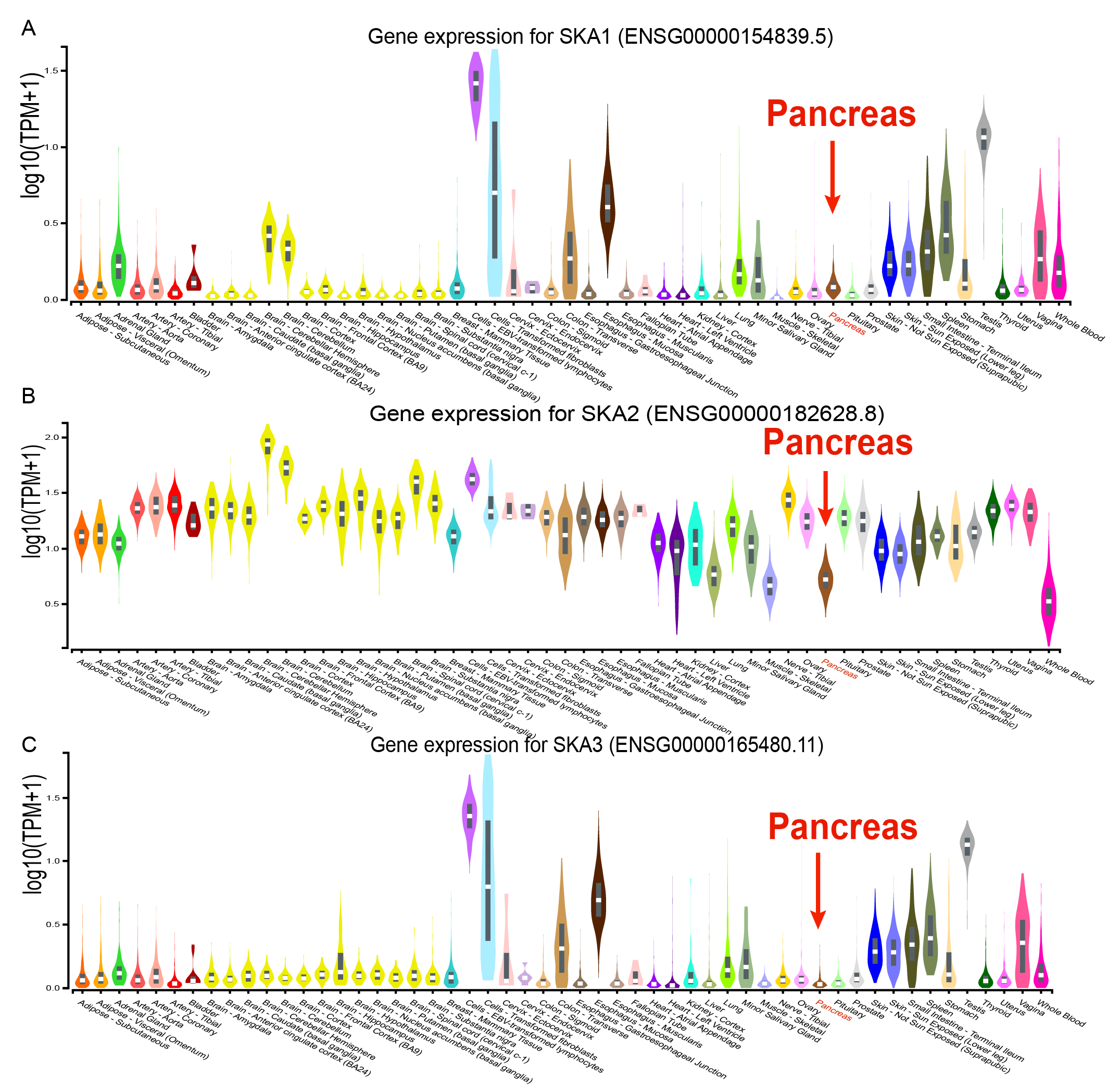

Supplement: Supplementary Figure 2 — The distribution of (A) SKA1, (B) SKA2, (C) SKA3 genes in human normal organ tissues. [file Image_2.TIF]

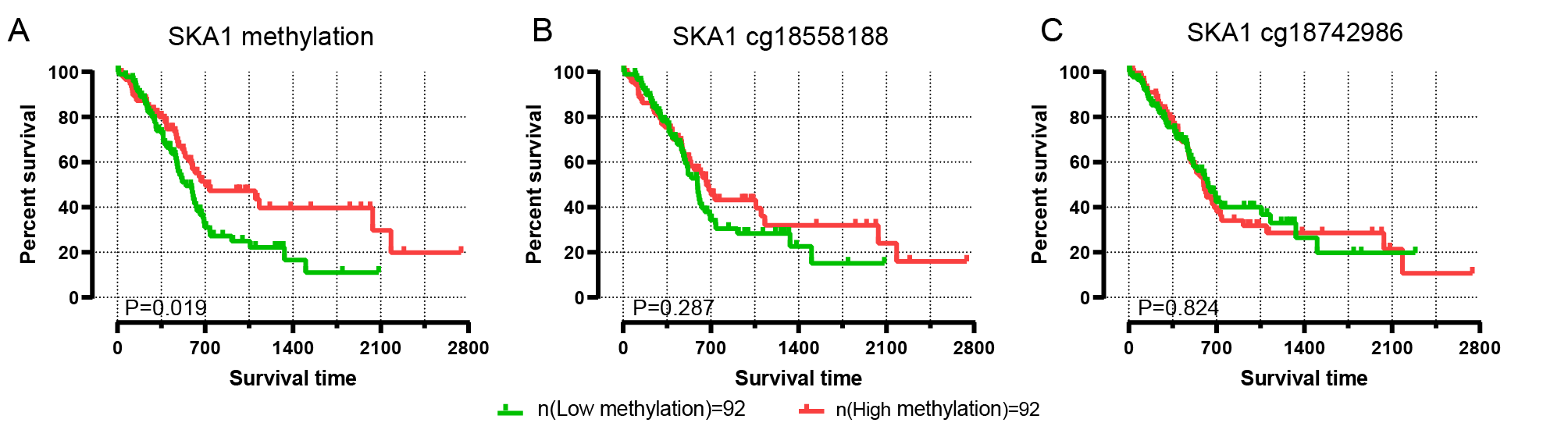

Supplement: Supplementary Figure 3 — Kaplan-Meier survival curves showing the survival significance of SKA1 DNA methylation in pancreatic cancer patients. (A) Total methylation level of SKA1; (B) cg18558188; (C) cg18742986. [file Image_3.TIF]

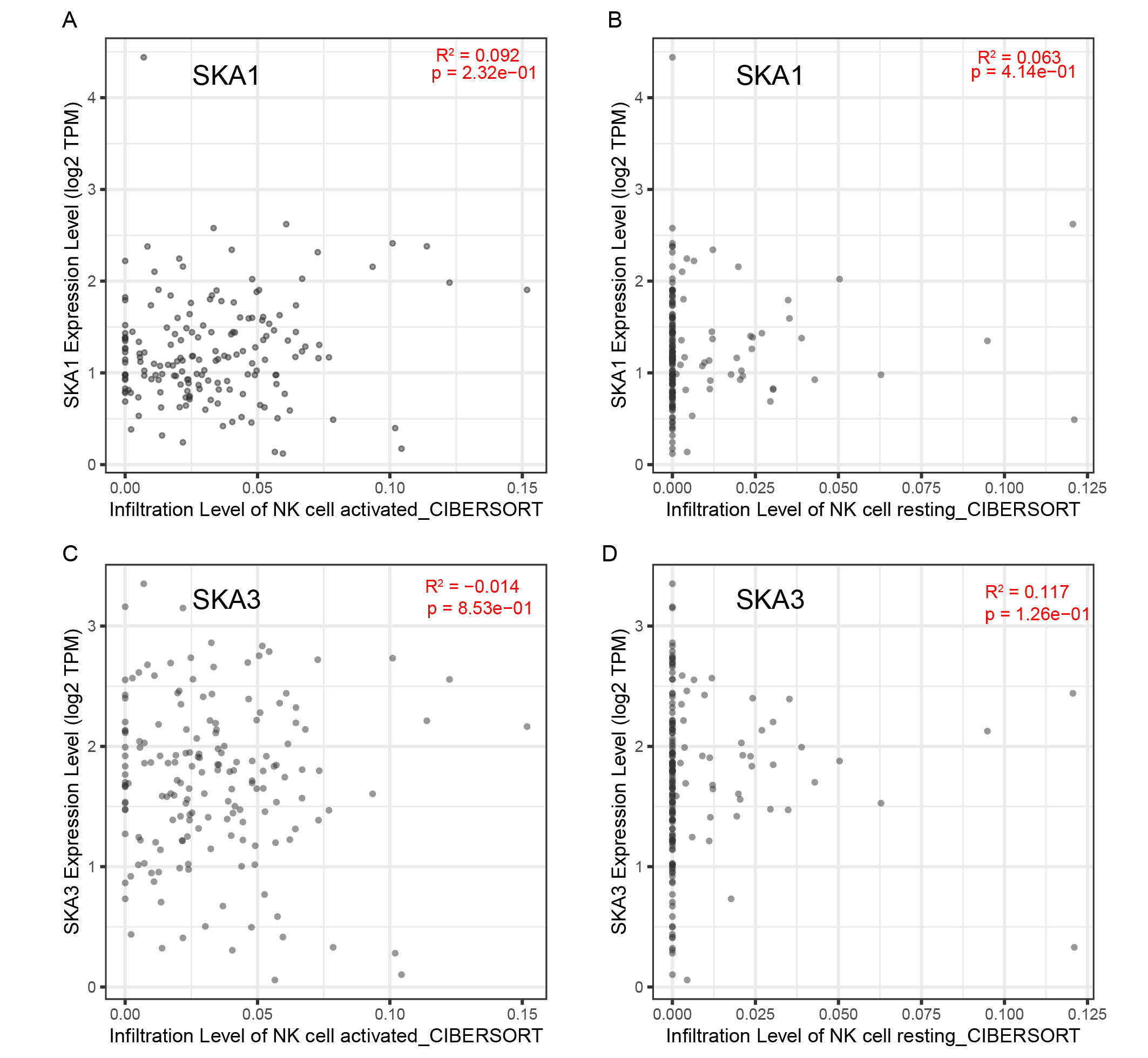

Supplement: Supplementary Figure 4 — The impact of SKA1 and SKA3 gene expression and mutation on tumour immunity. (A,B) SKA1 expression showed no correlation with infiltration levels of natural killer (NK) cells. (C,D) SKA3 expression is not associated with infiltration levels of natural killer (NK) cells. [file Image_4.TIF]
