# Supplementary material for: Identification of Spindle and Kinetochore-Associated Family Genes as Therapeutic Targets and Prognostic Biomarkers in Pancreas Ductal Adenocarcinoma Microenvironment
Source: Front Oncol. 2020 Nov 2;10:553536. doi: 10.3389/fonc.2020.553536 (PMC7667267; doi:10.3389/fonc.2020.553536)
Supplement: Supplementary Table 1 — Details of the GEO datasets included in this study. [file Table_1.DOCX]

**Supplementary Table 1.** Details of the GEO datasets included in this study.

| **Datasets** | **References** | **Year** | **Country** | **Platform** | **Sample size (tumor/control)** |
| --- | --- | --- | --- | --- | --- |
| GSE91035 | Schmittgen et al. | 2016 | USA | GPL22763 | 50 (27/23) |
| GSE101462 | Boerries et al. | 2017 | Germany | GPL10558 | 10 (6/4) |
| GSE1542 | Ishikawa et al. | 2004 | Japan | GPL96/GPL97 | 49 (24/25) |
| GSE74629 | None | 2015 | Spain | GPL10558 | 50 (36/14) |
| GSE71729 | Moffitt et al. | 2015 | USA | GPL20769 | 357 (145/46) |
| GSE62165 | Janky et al. | 2014 | Belgium | GPL13667 | 131 (118/13) |
| GSE62452 | Yang et al. | 2014 | USA | GPL6244 | 130 (69/61) |
| GSE28735 | Zhang et al. | 2011 | USA | GPL6244 | 90 (45/45) |
| GSE15471 | Badea et al. | 2009 | Romania | GPL570 | 78 (39/39) |
| GSE16515 | Pei et al. | 2009 | USA | GPL570 | 52 (36/16) |
| GSE32676 | Donahue et al. | 2011 | USA | GPL570 | 32 (25/7) |
