# Supplementary material for: Identification of Spindle and Kinetochore-Associated Family Genes as Therapeutic Targets and Prognostic Biomarkers in Pancreas Ductal Adenocarcinoma Microenvironment
Source: Front Oncol. 2020 Nov 2;10:553536. doi: 10.3389/fonc.2020.553536 (PMC7667267; doi:10.3389/fonc.2020.553536)
Supplement: Supplementary Table 2 — Correlations between prognosis-related gene and Gene Markers of cancer escape in TCGA and GSE62452. [file Table_2.DOCX]

| Markers | TCGA | | | | |  | GSE62452 | | | | |
| --- | --- | --- | --- | --- | --- | --- | --- | --- | --- | --- | --- |
|  | *SKA1* | |  | *SKA3* | |  | *SKA1* | |  | *SKA3* | |
|  | Cor | sig |  | Cor | sig |  | Cor | sig |  | Cor | sig |
| CD47 | 0.32 | *** |  | 0.34 | *** |  | 0.38 | ** |  | 0.44 | *** |
| PDL1 | 0.26 | *** |  | 0.25 | *** |  | 0.22 | ns |  | 0.35 | ** |
| B2M | 0.31 | *** |  | 0.23 | ** |  | 0.29 | * |  | 0.32 | ** |
| PD1 | 0.04 | ns |  | -0.12 | ns |  | -0.16 | ns |  | -0.09 | ns |
| CTLA4 | 0.01 | ns |  | 0.09 | ns |  | 0.09 | ns |  | 0.11 | ns |

**Supplementary Table 2.** Correlations between prognosis-related gene and Gene Markers of cancer escape in TCGA and GSE62452.

*, p < 0.05, **, p < 0.01, ***, p < 0.001.
